# Comparative Evaluation of the Sniffing Position with Simple Head Extension for Laryngoscopic View and Intubation Difficulty in Adults Undergoing Elective Surgery

**DOI:** 10.1155/2011/297913

**Published:** 2011-10-29

**Authors:** Smita Prakash, Amy G. Rapsang, Saurabh Mahajan, Shameek Bhattacharjee, Rajvir Singh, Anoop R. Gogia

**Affiliations:** ^1^Department of Anesthesia and Intensive Care, Vardhman Mahavir Medical College and Safdarjang Hospital, New Delhi 110029, India; ^2^Medical Research Centre, Hamad Medical Corporation (HMC), Doha 3050, Qatar

## Abstract

The effect of patient position on mask ventilation, laryngoscopic view, intubation difficulty, and the stance adopted by the anesthesiologist during laryngoscopy and tracheal intubation was investigated in 546 anesthetized adults in this prospective, randomized study. Patients were randomly assigned to either the sniffing position group or the simple extension group. The distribution of Cormack grades was comparable between the two groups (*P* = 0.144). The IDS score [median (IQR)] was 0 (0–2) in the sniffing group and 1 (0–2) in the simple extension group; *P* = 0.002. There were significant differences between groups with regard to intensity of lifting force, external laryngeal manipulation, alternate techniques used, number of attempts, and the stance adopted by anesthesiologist. We conclude that the sniffing position is superior to simple head extension with regard to ease of intubation as assessed by IDS. An upright stance is adopted by more anesthesiologists performing intubation with patients in the sniffing position.

## 1. Introduction

Maintenance of a patent airway is a fundamental responsibility of the anesthesiologist. Tracheal intubation remains one of the commonest means of establishing an airway. Placing the head and neck in the sniffing position has traditionally been considered important for obtaining good glottic visualization during direct laryngoscopy. The superiority of the sniffing position for laryngoscopy has been questioned [1, 2]. Adnet et al. [1] demonstrated that the sniffing position does not achieve alignment of the axes of the mouth, pharynx, and the larynx in awake subjects. They further reported that the sniffing position provides no advantage over simple head extension for improvement of glottic visualization except in obese and head extension-limited patients [2]. In their study, the complexity of intubation, as assessed by the Intubation Difficulty Scale (IDS), was found to be similar between patients intubated in either the sniffing position or the simple head extension position; however, data regarding individual variables of the IDS were not presented. Moreover, their study involved nonparalyzed patients. Laryngoscopy performed in the absence of neuromuscular blocking agents may be suboptimal. The objective of this prospective, randomized study was to determine the differences, if any, between the sniffing position and the simple head extension position with regard to the incidence of difficult mask ventilation and difficult laryngoscopy, intubation difficulty, and variables of the IDS, and in the stance adopted by the anesthesiologist performing laryngoscopy and tracheal intubation in adult patients undergoing elective surgery under general anesthesia with muscle paralysis. The study hypothesis was that the sniffing position would be superior to the simple head extension position for glottic visualization during direct laryngoscopy and would facilitate intubation. 

## 2. Materials and Methods

After obtaining approval by the local ethics committee and informed written consent, 550 consecutive ASA physical status I–III adult patients scheduled for elective surgical procedures requiring tracheal intubation were included in this prospective, randomized study. Patients with obvious malformation of the neck or face in whom tracheal intubation under general anesthesia would be contraindicated, unstable cervical spine, and patients requiring rapid sequence induction were excluded from the study. Preoperative airway evaluation was performed by one anesthesiologist involved in the study to avoid interobserver variability and included the following: (1) abnormal dentition: loose, protruding, or missing upper incisors or canines; (2) modified Mallampati classification as described by Samsoon and Young; [3] class I = soft palate, fauces, uvula, and pillars seen; class II = soft palate, fauces, and uvula seen; class III = soft palate and base of uvula seen; class IV: soft palate not visible; (3) temporo-mandibular joint mobility assessed by interincisor gap < or >3 cm and forward protrusion of the mandible (ability to move the lower teeth in front of the upper teeth; (4) thyromental distance and sternomental distance measured as the straight distance (approximated to the nearest 0.5 cm) from the thyroid notch and upper border of the manubrium sterni to the mentum, respectively, with the head in full extension and the mouth closed; (5) neck length measured as the straight distance from the mastoid process to sternal head of clavicle with head in neutral position; (6) the maximum range of neck and head movement <80° or >80° measured as described by Wilson et al. [4], wherein a pencil is placed vertically on the forehead of the patient with the head and neck in full extension. The patient is asked to fully flex while the change in angle is gauged by the anesthesiologist and classified as < or >80°; (7) body mass index, calculated as the weight (kg) divided by the square of the height (m); (8) other features such as the presence of a short neck, beard, or cervical spondylosis were noted. 

Each patient received oral alprazolam 0.25 mg/0.5 mg (< or >50 kg body weight, resp.) the night before and the morning of surgery. In the operating room, intravenous access and standard monitoring (electrocardiogram, pulse oximetry, capnography, and noninvasive blood pressure) was established. Before induction of anesthesia, patients were randomized into one of the two groups: the Sniffing group included supine patients intubated with head in the “sniffing position” by placing an incompressible head ring (height = 7 cm) under the head and extending the head on the neck; the Simple extension group included patients intubated with the head in simple extension without the head ring. Randomization (sealed envelopes) was done by a computer-generated random number table. The height of the operating table was adjusted such that the plane of the patient's face in both the groups was at the level of xiphisternum of the anesthesiologist performing laryngoscopy and intubation.

Anesthesia was induced with fentanyl (2 *μ*g/kg) and propofol (2–2.5 mg/kg) till loss of verbal contact. Intubation was facilitated by vecuronium 0.1 mg/kg. The patient's lungs were ventilated with oxygen and nitrous oxide (50 : 50) and isoflurane 0.6% for 3 min. Mask ventilation was graded as easy, difficult, or impossible [5]. Difficult mask ventilation was defined by one or more of the following criteria: inability for a unassisted anesthesiologist to maintain oxygen saturation greater than 90% using 100% oxygen and positive pressure mask ventilation; necessity to perform a two-handed mask; ventilation technique; important gas flow leak from the face mask, no perception of chest movements. Need for an oropharyngeal airway was also noted.

Laryngoscopy was performed using Macintosh size 3 blade by one of three authors, each having over four years experience in anesthesiology, and competent with respect to airway management. The laryngoscopic view was graded according to Cormack and Lehane grading scale [6]; grade (1) complete visualization of the vocal cords; grade (2) visualization of the inferior portion of the glottis; grade (3) visualization of only the epiglottis; and grade (4) nonvisualized epiglottis. No external laryngeal pressure was applied for grading the laryngoscopic view. Difficult laryngoscopy was defined as Cormack and Lehane grade 3 or 4. External laryngeal manipulation (bimanual laryngoscopy) was permitted, if necessary, after evaluation of laryngoscopy grade to facilitate the insertion of the tracheal tube. The laryngoscopic view obtained following external laryngeal manipulation (ELM) was also noted. 

Intubation was performed with tracheal tube size 7 in women and size 8 in men.

Intubation difficulty was assessed by the Intubation Difficulty Scale as described by Adnet et al. [7]. This quantitative scale is based on seven variables (number of tracheal intubation attempts; number of operators attempting intubation; number of alternative techniques used; glottis exposure as defined by Cormack and Lehane grade; subjective assessment of intensity of lifting force applied during laryngoscopy; need for external laryngeal manipulation; position of the vocal cords). Alternative techniques included repositioning of the patient, change of blade or tube, addition of a stylet, change to nasotracheal intubation, or use of fibroscopy or intubating laryngeal mask airway [7]. The IDS score was calculated in each case. A score of 0 represents an ideal intubation with minimum difficulty, an IDS score between 1 and 5 represents slight difficulty, and an IDS score greater than 5 represents moderate to major difficulty [7]. 

Duration of laryngoscopy (defined as the time from the instant the laryngoscope blade touched the patient until tracheal intubation and removal of the laryngoscope blade from the mouth) was noted. Laryngoscopy was considered prolonged if its duration exceeded 15 s. The stance of the anesthesiologist performing laryngoscopy and intubation (upright or leaning backwards, bending at the knee, or stooping to bring the face closer to the patient) was noted by an independent observer, not otherwise involved in the study. 

### 2.1. Statistical Analysis

The incidence of difficult laryngoscopy in a previous study was found to be 11.4% and 10.7% in the sniffing and simple extension positions, respectively [2]. Using this incidence of difficult laryngoscopy as the primary outcome measure, an estimated sample size of 42438 patients per group would be required with use of *α* = 0.05 and *β* = 0.10. As a trial of this size would require considerable time and resources, we chose a convenience sample of 550 patients. The primary outcome measure was the incidence of difficult laryngoscopy (Cormack grade 3 or 4). Secondary outcome measures included IDS score, variables of the IDS score, and the stance adopted by the anesthesiologist performing laryngoscopy and intubation.

Descriptive statistics were calculated for continuous variables as mean, standard deviation (mean ± SD), median and interquartile range (IQR) and for categorical variables as frequency distribution and percentage (*n* [%]). Student's unpaired *t*-test (for continuous variables) and chi-square test with Yate's correction factor (for categorical variables) were used to see the significance difference between the groups. *P* < 0.05 was regarded as statistically significant. SPSS 14.0 for Windows statistical software (SPSS Inc., Chicago, IL, USA) was used for statistical analysis. 

## 3. Results

A total of 550 consecutive adult patients were enrolled in the study. Four patients in the simple extension group were excluded because of nonstandardized intubating conditions. Thus, data from 546 patients was analyzed. The baseline characteristics are presented in Table 1. Compared with the simple extension group, patients in the sniffing group were older (*P* = 0.022), heavier (*P* = 0.022), and had a greater BMI (*P* = 0.005). The groups were comparable with regard to the presence or absence of factors predictive of difficult intubation (Table 1). There was no instance of impossible mask ventilation or failed intubation. One patient in simple extension group with Cormack grade 2 view at laryngoscopy had esophageal intubation. 

The incidence of difficult mask ventilation was 6.9% and 5.9% in the sniffing and simple extension groups, respectively (*P* = 0.632). The duration of laryngoscopy was comparable (14.2 ± 7.6 s in sniffing group and 13.9 ± 6.2 s in simple extension group; *P* = 0.639).

The distribution of Cormack and Lehane grades was comparable between the two groups. *P* = 0.144 (Table 2). The incidence of difficult laryngoscopy (defined as Cormack and Lehane grade 3 or 4) was 8% (22 of 275 patients) in the sniffing group and 9.2% (25 of 271 patients) in the simple extension group; *P* = 0.610. While external laryngeal manipulation improved Cormack grades in both groups, the improvement was more marked in the simple extension group; *P* = 0.04 (Table 3). 

**Figure 1:**
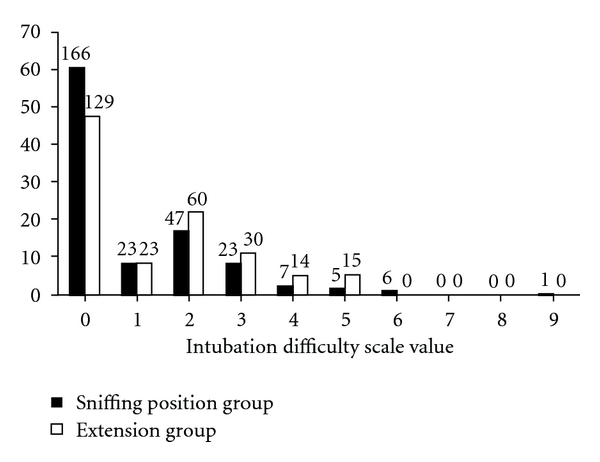
The distribution of Intubation Difficulty Scale scores among patients in the sniffing position and the simple head extension group. The absolute numbers of patients is shown above each vertical bar.

The distribution of IDS scores for patients in the two groups is shown in Table 4 and Figure 1. The IDS values ranged from 0 to 9. The median (25th–75th percentile) was 0 (0–2) in the sniffing group and 1 (0–2) in the simple extension group (*P* = 0.002). The details of individual variables of the IDS score and the alternative techniques used are presented in Tables 5 and 6. A stylet was used in 4.4% (12/275) of patients in the sniffing group compared with 8.9% (24/271) of cases in the simple head extension group; *P* = 0.034.

There was a statistically significant difference between the two groups in the stance adopted by the anesthesiologists performing laryngoscopy and intubation; in 71.2% (193 of 271 patients) of cases in the simple extension group, the anesthesiologist leaned backwards, bent at the knee, or stooped to bring the face closer to the patient during laryngoscopy and intubation to obtain the best laryngeal view compared with 14.9% (41 of 275 patients) in the sniffing position (*P* < 0.001).

## 4. Discussion

The results of this study indicate that there are no significant differences in the degree of glottic visualization obtained during direct laryngoscopy with a Macintosh blade in anesthetized and paralyzed patients in the sniffing position or the simple head extension position. A significantly greater number of intubations were judged to be easy (IDS score = 0) in patients intubated in the sniffing position compared with those intubated in simple head extension. The stance adopted by the anesthesiologists performing laryngoscopy and tracheal intubation was upright in a greater number of patients intubated in the sniffing position compared to that in simple head extension. 

In a series of 456 patients undergoing tracheal intubation under general anesthesia without muscle paralysis, Adnet et al. [2] reported that the sniffing position offered no advantage over simple head extension for improvement of glottic visualization, except in obese and head extension-limited patients. Our results are comparable with those described by Adnet et al. [2] with regard to laryngoscopic view obtained in the two positions, but are contrary with regard to intubation difficulty as determined by the IDS score. Glottic exposure alone is not completely representative of intubation difficulty. In our study, a significant number of patients intubated in the simple head extension position required more than one attempt at intubation, external laryngeal manipulation (ELM), use of alternate techniques, and use of increased force during laryngoscopy compared to those intubated in the sniffing position. Cormack grade 1 laryngoscopic view is not necessarily associated with an easy intubation [7]. A greater number of patients in the simple extension group with Cormack grade 1 view required external laryngeal manipulation compared with the sniffing position. This was because the tracheal tube tended to slip posterior to the posterior commissure. The use of optimal ELM can improve the laryngoscopic view considerably, and, in some patients, may make the difference between intubation failure and success [8]. Of interest is that ELM, when required, resulted in greater improvement in Cormack grades in the simple extension group compared with the sniffing position group. The degree of glottic exposure to an extent depends on the intensity of effort during the laryngoscopy. In our study a significantly greater number of patients in the simple head extension position (12.9%) required subjectively assessed greater lifting force during laryngoscopy compared with those in the sniffing position (5.8%). In the sniffing position, less of the tongue obscures the view of the larynx, and consequently there is less need for strenuous effort to displace the tongue anteriorly [9]. 

Previous studies have demonstrated improved laryngeal exposure by increasing head and neck elevation [10–12]. Schmitt and Mang [10] suggested that elevation of the head and neck beyond the sniffing position may improve visualization of the glottic structures in case of difficult laryngoscopy, leading to better intubation performance. Laryngeal exposure during laryngoscopy is better in the 25° back-up position compared to the flat supine position as demonstrated by significantly improved percentage of glottic opening scores [11]. Increasing head elevation and neck flexion significantly improved percentage of glottic opening scores during laryngoscopy on fresh human cadavers [12].

It is widely believed that in the sniffing position the oral, pharyngeal, and laryngeal axes are brought more nearly into a straight line [13–15]. Adnet et al. [1], using magnetic resonance imaging, found that it is not possible to achieve anatomic alignment of the laryngeal, pharyngeal, and the mouth axes in the neutral, simple head extension, or the sniffing position. However, the angle between the line of vision and the laryngeal axis decreased in both the simple head extension and the sniffing position compared with the neutral position. In addition, the sum of the angle between the pharyngeal axis and the laryngeal axis and that between mouth axis and pharyngeal axis was the least in the sniffing position, suggesting an advantage of the sniffing position over simple head extension position. This study involved nonanaesthetized volunteers, and the laryngoscope blade was not used.

Placing the patient in the sniffing position does not align the anatomic airway axes, and application of a force via the laryngoscope blade is required to achieve this [16]. Candido et al. [17] describe the axes in terms of laryngoscopic mouth axis, laryngoscopic pharyngeal axis, and laryngoscopic laryngeal axis which may be aligned to produce a laryngoscopic line (defined as a straight line passing through the inferior extremity of the superior incisors and the centre of the vocal cords). The anterior and caudad force exerted by a laryngoscope blade on the oropharyngeal structures with the head in the sniffing position displaces the soft tissues of the oropharyngeal cavity via the conversion of a potential space and also aligns the laryngoscopic axes resulting in visualization of the vocal cords [17]. It is the alignment of the laryngoscopic axes rather than the anatomic axes that is clinically relevant during tracheal intubation. 

There were four patients, all in the sniffing position group, in whom IDS exceeded 5. Two patients in the sniffing position needed to be repositioned to simple extension by removal of the head ring to facilitate intubation. This suggests that while the sniffing position may allow for easier intubation in a majority of patients, there may be a subset of patients in whom simple head extension or hyperextension with a shoulder roll may be necessary to perform intubation. 

Although the importance of correct positioning of the head and neck during laryngoscopy and intubation has been emphasized, there is paucity in the literature regarding the posture to be used while intubating the trachea. Because the patient's face is lower when simple extension position is used compared with the sniffing position, the height of the table was adjusted such that the patient's facial plane was at the level of the xiphisternum of the anesthesiologist performing laryngoscopy and intubation. The anesthesiologists in the sniffing position group adopted an erect posture in 85% of cases. In contrast, in 71% of cases in the simple head extension group, the anesthesiologists either bent at their knees, stooped, or leaned their upper body backwards. This suggests that alignment of the line of vision and the laryngeal axis is better in the sniffing position compared with the simple head extension position. The fact that stooping or bending was required during intubation in patients in simple extension position, in spite of similar Cormack-Lehane scores, also suggests that the standardized height of table with face level of patient with xiphisternum of the operator may not be ideal for the simple extension position. 

Our findings are clinically relevant. Repeated tracheal intubation attempts may contribute to patient morbidity. The incidence of airway and hemodynamic complications increases beyond two laryngoscopic attempts during emergency airway management in the remote location [18]. Suboptimal laryngoscopy may result in accidental esophageal intubation which in the emergency setting can have grave consequences. Most patients, especially the elderly, are more comfortable with occipital support compared to lying flat on the operating table. Since it is easier to remove a pillow placed *a priori* under the patient's head than to attempt to place one under the head should the indication arise, then it is logical to commence laryngoscopy with a pillow placed under the patient's occiput [19]. 

An important limitation of this study is failure to blind observers. Due to obvious differences in head position, we were unable to devise a method in which investigators were unaware of group allocation. In our study, the authors were also the operators. Involvement of other operators blinded to the purpose of the study could have limited bias towards any one position. In addition, the study involved three operators whose intubations have been aggregated for statistical analysis. It is possible that the differences in stance of the anesthesiologist could be operator dependent. Unfortunately, our data acquisition does not allow for this determination.

It is important to note that our findings apply only when a curved laryngoscope blade (Macintosh) is used. When otolaryngologists perform direct laryngoscopy, or rigid bronchoscopy, the neck is hyperextended by placing a roll under the shoulders in order to accentuate neck extension. This allows them to get a straight line of vision from the mouth into the trachea. To our knowledge, no previous study has demonstrated any disadvantage of the sniffing position as a head position for tracheal intubation; rather, the sniffing position has been found to be advantageous in obese and head extension-limited patients [2]. Our results emphasize the importance of the sniffing position for optimal laryngeal exposure and ease of intubation. Whilst glottic visualization was similar in patients placed in the sniffing or the simple head extension position, the sniffing position conferred a distinct advantage for tracheal intubation as assessed by the IDS. We conclude that, compared to the simple head extension position, the sniffing position should be used as a standard head position before intubation attempts under general anesthesia.

##  Funding 

The authors received institutional funding.

## Figures and Tables

**Table 1:** Patient characteristics.

	Sniffing position	Simple extension	*P* value
	(*n* = 275)	(*n* = 271)	
Age (yr)	38.7 ± 13.1	36.1 ± 14.1	0.022
Sex ratio (M : F)	80 : 195	94 : 177	0.161
Weight (kg)	54.7 ± 11.1	52.8 ± 8.5	0.022
Body mass index (kg/m^2^)	21.5 ± 4.1	20.6 ± 3.3	0.005
Thyromental distance (cm)	7.1 ± 0.8	7 ± 0.8	0.118
Sternomental distance (cm)	15 ± 1.7	14.9 ± 1.6	0.705
Neck length (cm)	15.7 ± 1.6	15.7 ± 1.7	0.840
*Predictors of difficult intubation*			
Mouth opening <3 cm	0 (0)	0 (0)	—
Modified Mallampati class^3^ I/II/III/IV	176/56/22/21	181/44/31/15	0.260
Thyromental distance <6.5 cm	37 (13.5)	45 (16.6)	0.303
Sternomental distance <12.5 cm	12 (4.4)	10 (3.7)	0.689
Neck movement <80°	0 (0)	0 (0)	—
Short neck	14 (5.1)	11 (4.1)	0.683
Receding mandible	3 (1.1)	1 (0.4)	0.624
Beard	4 (1.5)	7 (2.6)	0.380
Maxillary incisors			0.582
Loose	4 (1.5)	4 (1.5)	
Missing	10 (3.6)	7 (2.6)	
Protruding	17 (6.2)	16 (5.9)	
Edentulous	8 (2.9)	3 (1.1)	

Values are mean ± SD or numbers (percent).

**Table 2:** Comparison of views obtained during laryngoscopy.

Cormack and Lehane	Sniffing position	Simple extension	*P* value
	(*n* = 275)	(*n* = 271)	
Grade 1	171 (62.2)	145 (53.5)	0.144
Grade 2	82 (29.8)	101 (37.3)	
Grade 3	21 (7.6)	25 (9.2)	
Grade 4	1 (0.4)	0 (0)	

Values are numbers (percent).

**Table 3:** Comparison of views obtained during laryngoscopy following external laryngeal manipulation.

Cormack and Lehane	Sniffing position	Simple extension	*P* value
	(*n* = 90)	(*n* = 118)	
Grade 1	57 (63.3)	92 (78.0)	0.04
Grade 2	32 (35.6)	26 (22.0)	
Grade 3	1 (0.01)	0 (0)	

Values are numbers (percent).

**Table 4:** Intubation Difficulty Scale (IDS) score.

IDS score	Sniffing position	Simple extension	*P* value
	(*n* = 275)	(*n* = 271)	
0	166 (60.4)	129 (47.6)	0.005
0–5	105 (38.2)	142 (52.4)	
>5	4 (1.5)	0 (0)	

Values are numbers (percent).

**Table 5:** Variables of the intubation difficulty scale.

	Sniffing position	Simple extension	*P* value
	(*n* = 275)	(*n* = 271)	
Attempts > 1	20 (7.3)	34 (12.5)	0.010
Operators > 1	0 (0)	0 (0)	—
Cormack grade > 1	104 (37.8)	126 (46.5)	0.610
Increased lifting force	16 (5.8)	35 (12.9)	0.004
ELM	90 (32.7)	118 (43.5)	0.009
Alternative techniques	13 (4.7)	26 (9.6)	0.027
Vocal cords adducted	0 (0)	0 (0)	—

ELM: External laryngeal manipulation. Values are numbers (percent).

**Table 6:** Alternate techniques used.

	Sniffing position	Simple extension	*P* value
	(*n* = 13)	(*n* = 26)	
Repositioning	2 (15.4)	1 (3.8)	0.027
Change of blade	0 (0)	0 (0)	
Change of tube	1 (7.7)	1 (3.8)	
Use of stylet	12 (92.3)	24 (92.3)	0.034
Intubating LMA	1 (7.7)	0 (0)	

Values are numbers (percent).
